# Therapeutic Management of Vestibular Disorders During Pregnancy: A Narrative and Evidence-Based Review

**DOI:** 10.7759/cureus.89705

**Published:** 2025-08-09

**Authors:** Melissa Castillo-Bustamante, Anita Bhandari, Neşe Çelebisoy, Susan L Whitney, Michelle R Petrak, Maria N Campo-Campo

**Affiliations:** 1 Otolaryngology, Clinica Universitaria Bolivariana, Medellín, COL; 2 School of Medicine, Health Sciences School, Universidad Pontificia Bolivariana, Medellín, COL; 3 Otolaryngology, Mahatma Gandhi Institute of Medical Sciences and Technology, Jaipur, IND; 4 Otolaryngology, Neuroequilibrium, Jaipur, IND; 5 Department of Neurology, Ege University Medical School, Izmir, TUR; 6 Physical Medicine and Rehabilitation, University of Pittsburgh, Pittsburgh, USA; 7 Audiology, Interacoustics, Middelfart, DNK; 8 Audiology, Northwest Speech and Hearing, Arlington Heights, USA; 9 Maternal-Fetal Medicine, Clinica Universitaria Bolivariana, Medellin, COL

**Keywords:** dizziness diagnosis, pregnancy, vestibular assessment, vestibular disorders, vestibular testing

## Abstract

Vestibular disorders such as vestibular migraine (VM), benign paroxysmal positional vertigo (BPPV), Ménière’s disease (MD), persistent postural-perceptual dizziness (PPPD), and vestibular neuritis (VN) are not uncommon among women of reproductive age and may occur or worsen during pregnancy. However, the management of these disorders during gestation is complicated by physiological changes, the limited safety data on many medications, and ethical constraints in clinical research involving pregnant individuals. This review focuses on the therapeutic strategies employed in managing vestibular disorders during pregnancy, highlighting pharmacological and non-pharmacological interventions, evidence-based approaches, current limitations, and areas requiring further research. Emphasis is placed on safety profiles, individualized care, and interdisciplinary collaboration to optimize maternal health while minimizing fetal risk.

## Introduction and background

Vestibular disorders are a significant source of morbidity among women of reproductive age, often affecting balance, spatial orientation, and quality of life [1]. Among the most prevalent of these disorders are vestibular migraine (VM), benign paroxysmal positional vertigo (BPPV), Ménière’s disease (MD), persistent postural-perceptual dizziness (PPPD), vestibular neuritis (VN), and bilateral vestibulopathy (BVP) [2]. Vestibular disorders in pregnant women have a global prevalence of approximately 32 per 100,000 individuals [3]. While each of these conditions presents with distinct pathophysiological mechanisms and clinical profiles, they share a common challenge when occurring during pregnancy: the need to manage disabling symptoms in a setting where pharmacologic options are limited, diagnostic clarity may be impaired, and maternal-fetal safety must be paramount [3].

Pregnancy induces profound physiological changes-from hormonal shifts and vascular adaptations to biomechanical alterations-that can directly or indirectly affect vestibular function [4]. During advanced pregnancy, women may be at higher risk for falling during ambulation in the dark [5]. Elevated levels of estrogen and progesterone modulate neurotransmission, endolymphatic fluid dynamics, and vascular reactivity within the inner ear [6]. Simultaneously, changes in blood pressure regulation, blood volume expansion, and altered proprioception may enhance susceptibility to vertigo or dizziness, particularly in patients with underlying vestibular pathology [7]. However, these same physiological transitions may also confound clinical assessment, as many of the symptoms of vestibular disorders - such as nausea, lightheadedness, or unsteadiness - overlap with normal gestational phenomena, especially during the first and third trimesters [8].

Despite their prevalence and potential severity, vestibular disorders in pregnancy remain underrecognized and undertreated [9]. The exclusion of pregnant individuals from randomized clinical trials and longitudinal vestibular research has led to a significant gap in evidence-based management strategies tailored to this population [10]. Most current recommendations are extrapolated from non-pregnant cohorts or derived from limited observational studies, case reports, or pharmacovigilance registries [11]. Furthermore, therapeutic hesitancy is common among clinicians due to the teratogenic potential of several vestibular-active medications and the ethical complexities inherent in balancing maternal symptom relief with fetal risk mitigation [11]. This narrative review explores the therapeutic and rehabilitative management of vestibular disorders during pregnancy, with an emphasis on practical considerations for clinical care. It discusses both pharmacological and non-pharmacological strategies, taking into account the specific challenges that arise in each trimester.

## Review

Therapeutic management of vestibular disorders during pregnancy: a narrative and evidence-based review

This narrative review was based on a non-systematic literature search using PubMed, Scopus, and Google Scholar. Keywords included “vestibular migraine”, “vertigo in pregnancy”, “Ménière’s disease pregnancy”, “benign paroxysmal positional vertigo (BPPV)”, “unilateral vestibulopathy”, “bilateral vestibulopathy”, “persistent postural-perceptual dizziness (PPPD)”, “pregnancy”, “vestibular disorders”, and “vestibular rehabilitation”. English-language articles published from 2000 to 2024 were considered. Selection favored clinical studies, reviews, and guidelines relevant to pregnancy-specific management.

Pregnancy is a dynamic physiological state marked by systemic modifications that influence the function of multiple organ systems, including those implicated in vestibular homeostasis [12]. Hormonal, hemodynamic, neurochemical, and metabolic shifts across the three trimesters not only predispose patients to vestibular disturbances but also modulate the natural history of pre-existing vestibular conditions [13]. Furthermore, the therapeutic landscape in pregnancy is uniquely constrained by fetal developmental considerations and evolving maternal physiology [12,13]. Understanding these trimester-specific interactions is critical to delivering safe and effective care for pregnant individuals presenting with vestibular symptoms [12,13].

In the first trimester, the maternal body undergoes abrupt hormonal and neurovascular changes aimed at establishing and supporting early gestation [14]. The sharp rise in human chorionic gonadotropin (hCG), progesterone, and estrogen modulates vascular tone, osmotic gradients, and neurotransmitter dynamics [14]. These hormonal shifts, particularly elevated estrogen levels, affect the inner ear by altering ionic composition in the endolymphatic fluid and influencing permeability of the stria vascularis, with downstream effects on vestibular signal transduction [15]. In parallel, central nervous system excitability is altered through serotonergic and GABAergic pathways, which may precipitate or worsen vestibular migraine attacks, especially in women with a prior history of migraine or migraine equivalents [16]. The increased fluid retention and alterations in calcium homeostasis also contribute to a milieu conducive to otoconia destabilization, which may explain the higher incidence of BPPV in some pregnant women during early gestation [17]. Functional disorders such as PPPD may also be triggered in this context, particularly in individuals experiencing heightened psychological vulnerability or neurophysiological dysregulation [18].

Clinically, the first trimester presents profound diagnostic ambiguity [19]. Symptoms such as nausea, lightheadedness, unsteadiness, and orthostatic intolerance, commonly seen in early pregnancy, closely mimic the presentation of true vestibular pathology, leading to potential delays in diagnosis or misattribution to gestational changes [19]. From a therapeutic perspective, this trimester is also the most restrictive, given that embryonic organogenesis occurs between the third and 10th weeks [20]. Teratogenic risk is at its peak, and pharmacologic agents are used sparingly, if at all [20]. Systemic corticosteroids are generally avoided due to associations with cleft palate in animal and limited human studies [21]. Selective serotonin reuptake inhibitors (SSRIs), although not contraindicated, should be reserved for severe or functionally disabling cases of PPPD or when comorbid mood disorders are present. In pregnant patients, their use must be carefully coordinated with the obstetrician-gynecologist and only under psychiatric supervision [22,23]. Even when pharmacologic treatment is deemed necessary, it is critical to prioritize the lowest effective dose and to weigh maternal benefit against potential fetal risks. Close follow-up, interdisciplinary communication, and clear documentation are essential to ensure both safety and therapeutic efficacy in this vulnerable stage of pregnancy [24].

The second trimester offers a comparatively stable physiological environment [25]. By this stage, the placenta assumes endocrine dominance, leading to a more gradual and predictable hormonal profile [25]. Cardiovascular adaptation becomes more robust, with increased plasma volume, reduced systemic vascular resistance, and improved cerebral perfusion [25-27]. These adaptations may result in partial symptom resolution in patients with vestibular migraine or PPPD; however, others may continue to experience episodic vertigo or chronic instability [26]. Notably, this is often the period when delayed diagnoses are made for vestibular disorders that were masked during the first trimester [27]. Furthermore, certain conditions, such as Ménière’s disease or bilateral vestibular hypofunction, may worsen in this phase due to salt retention, immune modulation, or cumulative vestibular decompensation [27].

From a therapeutic standpoint, the second trimester represents a therapeutic window of opportunity [27,28]. The relative stability in maternal physiology and the reduced teratogenic risk enable more aggressive, yet still cautious, intervention [28]. Vestibular rehabilitation therapy is well tolerated in most patients, with improved compliance and physical capacity [29]. Positional maneuvers for BPPV - such as the Epley or modified Semont maneuver - can be performed more comfortably than in later stages and may require minimal adaptation [29]. This trimester also allows for the careful introduction of pharmacologic therapies when non-pharmacological measures are insufficient [30]. SSRIs, particularly sertraline and escitalopram, may be introduced for PPPD or comorbid anxiety and depressive symptoms, with growing data supporting their safety when initiated in the second trimester [30]. Betahistine is generally not recommended during pregnancy due to the limited availability of human safety data [31]. Although animal studies have not shown direct fetal harm, the absence of robust human trials prevents definitive conclusions regarding its teratogenic potential [31]. As such, betahistine should not be initiated during pregnancy [31]. Inflammatory vestibular conditions requiring short-term corticosteroids, such as vestibular neuritis, may also be considered in select cases, though still approached with caution [32]. Overall, this trimester offers the most therapeutic flexibility and is an optimal time for initiating or intensifying individualized rehabilitation, psychological therapy, and multidisciplinary coordination [33].

The third trimester introduces a new spectrum of physiological challenges [4]. Rapid uterine growth increased fetal weight, and ligamentous laxity may alter the maternal center of gravity and proprioceptive feedback, leading to a higher incidence of gait instability and imbalance, even in healthy pregnancies [4]. In patients with underlying vestibular dysfunction, these changes may exacerbate chronic unsteadiness, oscillopsia, or postural fear [8]. Visual dependence often increases in patients with bilateral vestibulopathy or PPPD, compounding functional limitations and psychological distress [8]. The development of supine hypotensive syndrome due to uterine compression of the inferior vena cava further limits the feasibility of diagnostic maneuvers or rehabilitative techniques that require prolonged supine positioning [34].

Pharmacologic options become more constrained again during this trimester, particularly due to concerns about neonatal effects [35]. Benzodiazepines are contraindicated due to risks of neonatal withdrawal, hypotonia, and sedation [36]. Systemic corticosteroids such as dexamethasone or betamethasone may be indicated in specific vestibular scenarios but must be weighed against obstetric indications for fetal lung maturation protocols [37]. Vestibular suppressants may be cautiously used for short-term symptomatic relief but are discouraged for chronic management [38]. Additionally, rising maternal anxiety about childbirth, labor, and perinatal outcomes may amplify functional symptoms in patients with PPPD, necessitating integrated obstetric-psychiatric care [38].

Rehabilitation must be adapted to minimize fall risk, incorporating seated or supported balance exercises, dynamic gaze training within a safe range, and home safety counseling [39]. In this context, psychological interventions, including cognitive behavioral therapy (CBT), play a central role in promoting maternal resilience, reducing anticipatory anxiety, and mitigating symptom amplification [40].

Vestibular assessment and physical examination in pregnant patients with dizziness and vertigo

The physical examination plays a central role in the diagnostic approach to dizziness and vertigo during pregnancy, typically conducted by otolaryngology and/or neurology, with the support of multidisciplinary teams including physiotherapy and audiology, and the fundamental involvement of gynecology and obstetrics [9]. Given the high prevalence of nonspecific symptoms such as lightheadedness and imbalance in this population, a targeted vestibular evaluation is essential to differentiate between physiological changes of pregnancy and underlying vestibular pathology [9].

A structured vestibular examination begins with careful observation of spontaneous and gaze-evoked nystagmus, assessment of ocular alignment, and evaluation of smooth pursuit and saccadic eye movements [9]. The head impulse test (HIT) remains a cornerstone in identifying peripheral vestibular hypofunction and can be safely performed in pregnant individuals [9]. Dynamic visual acuity testing may further support the identification of vestibulo-ocular reflex deficits [9].

Positional testing, particularly the Dix-Hallpike and supine roll tests, is fundamental for the diagnosis of BPPV, which is one of the most common vestibular disorders encountered during pregnancy [4,9]. These maneuvers are generally well-tolerated and do not pose a significant risk to the mother or fetus when performed with proper technique and patient support [4,9]. However, in advanced gestation, modified positioning or the use of side-lying alternatives may be necessary to accommodate uterine size and prevent supine hypotensive syndrome [4,9].

In addition to bedside testing, instrumented vestibular assessments such as videonystagmography, video head impulse testing (vHIT), vestibular-evoked myogenic potentials (VEMPs), electrocochleography and posturography can be selectively utilized to support diagnosis, particularly in cases where symptoms are severe, atypical, or refractory to initial management [9]. These tests, which do not involve ionizing radiation, are generally considered safe during pregnancy when clinically justified [9].

Integrating focused vestibular examination into the routine evaluation of pregnant patients with dizziness or vertigo enhances diagnostic accuracy, allows for timely and appropriate management, and helps prevent unnecessary investigations or interventions [9]. As pharmacological options are often limited during pregnancy, the identification of specific vestibular disorders through physical examination and targeted vestibular testing is a key component of effective and individualized care [9].

Vestibular migraine

Vestibular migraine represents one of the most prevalent causes of episodic vertigo in women of reproductive age [41]. It is characterized by recurrent vestibular symptoms lasting minutes to hours, often accompanied by photophobia, phonophobia, or a history of migraine headaches [42]. Pregnancy induces significant hormonal shifts - most notably a rise in circulating estrogen and progesterone - that modulate neurotransmitter systems, including serotonin and gamma-aminobutyric acid (GABA), both of which are implicated in the pathogenesis of migraine [43-45]. Studies have shown that approximately 50-70% of women with migraine without aura experience symptom improvement during pregnancy, particularly in the second and third trimesters which is ascribed to many factors including the rise of oestrogen and endogenous opioid levels, raising the pain threshold and absence of menstruation-related hormonal fluctuations, which are a major trigger for migraine attacks [45,46]

However, the course of vestibular symptoms is less predictable, and vestibular episodes may persist independently of headache, making treatment during gestation more complex [44].

Therapeutic management of VM in pregnancy prioritizes non-pharmacological strategies due to concerns about teratogenicity [46]. Migraine is commonly associated with triggers, including stress, fatigue, and fasting, which are also known to trigger VM. As lifestyle intervention has been reported to be beneficial, advice about sleep hygiene, exercise, avoidance of triggers like fasting, caffeine restriction, loud sounds, strong odors, and bright lights, seems appropriate in VM patients [47]. Vestibular rehabilitation therapy (VRT) has demonstrated efficacy in reducing the frequency and severity of vertigo episodes and is safe for use throughout pregnancy [48]. VRT should be tailored to the patient’s tolerance and functional limitations [48]. CBT, mindfulness-based stress reduction techniques, yoga, and relaxation are also recommended as adjuncts, particularly in patients with comorbid anxiety or persistent symptoms [47,48].

Regarding pharmacologic options, for the abortive treatment, there is insufficient evidence to recommend any specific pharmacological therapy for the termination of acute vertigo attacks in VM. Triptans are currently considered safe in pregnancy (in particular sumatriptan) and have a proven efficacy in aborting migraine headaches; their effectiveness for the vestibular symptoms is inconclusive [49,50]. Some antiemetics, including promethazine and meclizine, have shown acceptable safety profiles (FDA Category B) and can be used cautiously for associated nausea and vertigo [51].

Acetaminophen remains the first-line analgesic for acute migraine headaches and is considered safe in all trimesters [52]. Acetylsalicylic acid (ASA) in low doses (<100 mg/day), non-selective COX-inhibitors seem also safe, when selective COX2-inhibitors, ergots, and ergot alkaloids are contraindicated [53]. Prophylactic treatments present a greater challenge. Magnesium supplementation (e.g., 400-600 mg/day) has been associated with a reduction in migraine frequency and is generally safe during pregnancy [54]. Β-blockers (metoprolol and propranolol) are the first-line option [53]. Tricyclic antidepressants (TCA) are considered the safest second-line option when β-blockers are contraindicated or ineffective. Amitriptyline is preferred [53,55]. The serotonin norepinephrine reuptake inhibitor (SNRI) venlafaxine should be avoided during pregnancy [53]. Valproate and topiramate are contraindicated because of their major teratogenic effects [53]. Lamotrigine has a good safety profile compared with other antiepileptic drugs [53,56]. Calcium channel blockers should also be avoided [53,56].

Botulinum toxin for the treatment of chronic migraine is considered safe in pregnancy and is often continued [57]. However, the evidence for botulinum toxin to treat vestibular symptoms is currently very limited [58]. Longitudinal studies and controlled trials are needed to establish safe and effective protocols for managing VM in pregnant populations [46]. In the management of migraine during pregnancy, certain nutritional supplements with favorable safety profiles may be considered as preventive options [59]. 

Riboflavin (vitamin B2) and coenzyme Q10 have shown potential in reducing migraine frequency and intensity in non-pregnant populations, and are sometimes used during pregnancy due to their low teratogenic risk. Similarly, other supplements such as magnesium, vitamin D, and B-complex vitamins may be considered during pregnancy [59]. When patients are already on preventive treatments before conception, careful evaluation should be conducted to determine whether these can be safely continued [59]. Interdisciplinary coordination, especially involving obstetrics, is key to ensuring both maternal symptom control and fetal safety [59]. Although robust data in pregnant cohorts are limited, both supplements are generally well-tolerated and may offer a non-pharmacological alternative for selected patients under medical supervision [59]. 

Benign paroxysmal positional vertigo

BPPV is the most common peripheral vestibular disorder in adults and a frequent cause of vertigo during pregnancy, particularly in the second and third trimesters [60]. BPPV is characterized by transient episodes of vertigo triggered by changes in head position, resulting from dislodged otoconia migrating into the semicircular canals - most commonly the posterior canal - causing abnormal endolymph flow and erroneous signaling of angular head movements [60]. BPPV of the posterior canal has the highest incidence, followed by horizontal canal BPPV [60]. It has been reported that patients who were tested for BPPV early in the history had a higher incidence of horizontal canal involvement than those tested later [61]. While BPPV occurs across all age groups, pregnancy-specific physiological factors such as increased calcium demand for fetal bone development, altered vitamin D metabolism, and elevated parathyroid hormone-related protein (PTHrP) levels may contribute to otoconial instability [60]. Moreover, biomechanical and postural alterations such as prolonged supine positioning and compression of vertebral venous outflow can compound vestibular stress and increase the risk of BPPV in pregnant women [62,63].

**Figure 1:**
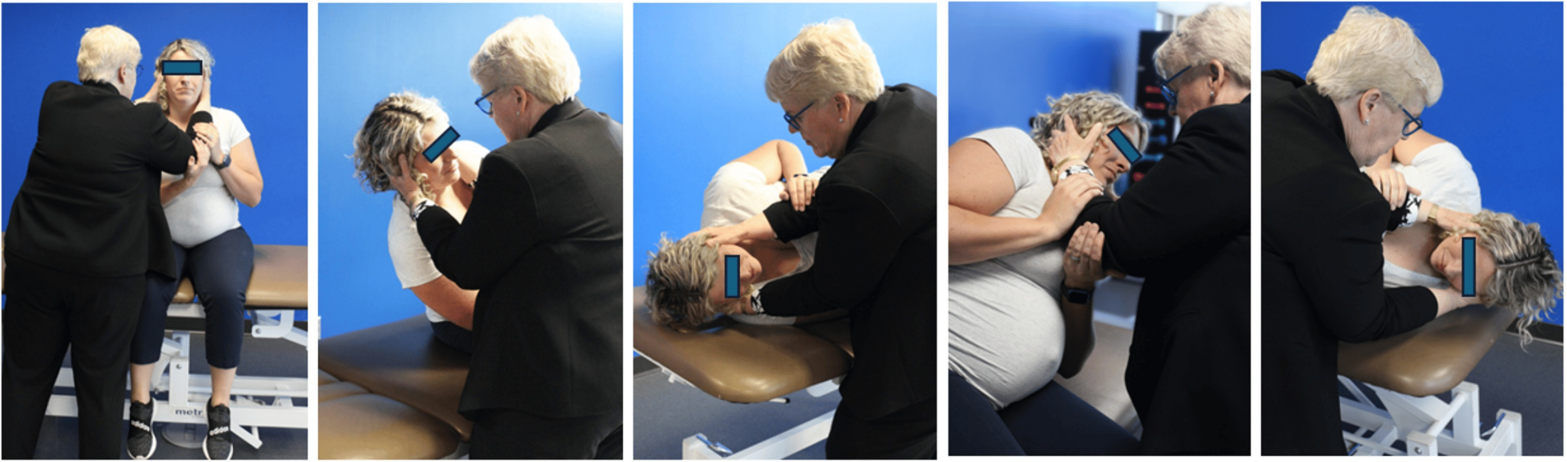
Li maneuver in a pregnant woman The figure has not been published in any other media or journals. Permissions obtained from the patient. Photo authored by Dr. Susan Whitney, co-author of this article, as a contribution to this publication.

The management of BPPV during pregnancy is primarily non-pharmacological and involves the use of repositioning maneuvers, which are both effective and safe [60]. The Epley maneuver is considered the gold standard for posterior canal BPPV. It is widely applied during pregnancy, although its execution may require modifications to accommodate the pregnant patient’s physical limitations [64]. For instance, care must be taken to avoid supine hypotension syndrome in the second and third trimesters by placing a wedge or pillow under the right hip or using lateral decubitus adaptations [35]. The Semont maneuver and Brandt-Daroff exercises serve as alternative therapeutic strategies, particularly in recurrent or bilateral cases [65]. The Zuma maneuver is useful for horizontal canal BPPV results, though its safety profile in pregnant populations remains underexplored [66]. The Li maneuver is ideal for women during the third trimester with posterior canal BPPV, as the patient sits on the edge of the bed while keeping the head forward throughout the maneuver [67]. They lie down to the involved side, then move 180 degrees to the non-involved side, then sit back up without any change of head position (Figure 1) [67].

**Figure 2:**
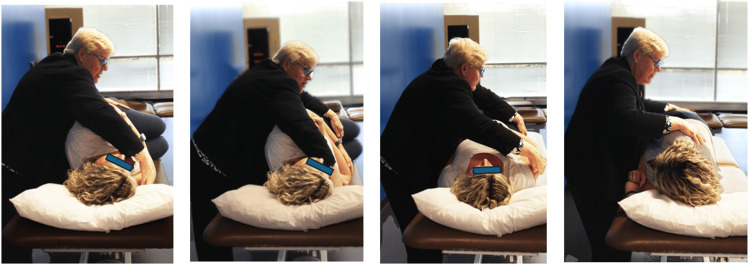
Li Quick maneuver in a pregnant woman The figure has not been published in any other media or journals. Permissions obtained from the patient. Photo authored by Dr. Susan Whitney, co-author of this article, as a contribution to this publication.

It is ideal for women who are experiencing back pain during their pregnancy [67]. The Li is as effective as the Epley maneuver [67]. The Li quick repositioning maneuver is ideal for women who have been diagnosed with horizontal canal canalithiasis (Figure 2) [67,68]. It required the person to roll to the involved side for one minute, to roll to the opposite side (180 degrees), and then sit up, making it a safe maneuver for persons who are pregnant [67,68]. The Li quick maneuver takes approximately three minutes to complete (Figure 2) [67,68]. 

**Figure 3:**
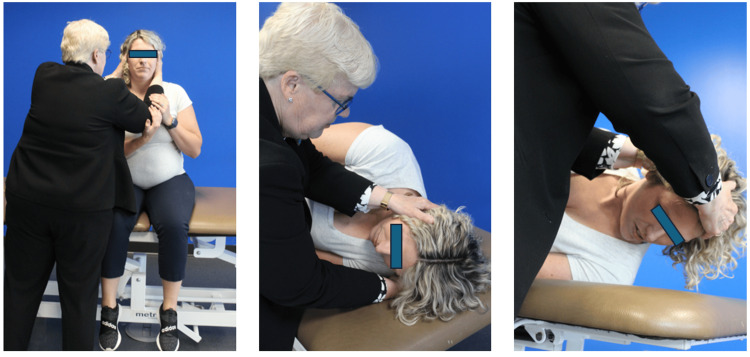
Guffoni maneuver in a pregnant woman The figure has not been published in any other media or journals. Permissions obtained from the patient. Photo authored by Dr. Susan Whitney, co-author of this article, as a contribution to this publication.

The Gufoni maneuver is considered a practical and safe option for the treatment of horizontal canal BPPV in pregnant patients. It avoids prolonged supine positioning, making it more comfortable in advanced gestation, and can be performed effectively on a standard examination table. Its simplicity and efficacy make it particularly suitable when modifications to traditional repositioning techniques are needed during pregnancy (Figure 3) [67,68]. 

Pharmacologic therapy is generally discouraged in the treatment of BPPV due to the mechanical rather than neurochemical etiology of the disorder [69]. However, short-term use of vestibular suppressants such as meclizine or dimenhydrinate may be considered in severe, incapacitating episodes associated with nausea or vomiting [69]. Both agents are classified as FDA Category B, indicating no known risk in animal studies but limited human data [69]. Importantly, these medications should be used with caution and only when the vertigo episodes are so debilitating that they impair maternal hydration, mobility, or sleep [69]. Long-term use is not recommended as it may hinder central vestibular compensation and adaptation [69].

Vestibular rehabilitation may be employed as an adjunct or in cases of persistent or recurrent symptoms [70]. Supervised VRT protocols focusing on habituation and balance retraining are particularly useful for women experiencing residual dizziness or those with comorbid balance impairments [70]. Clinicians should be aware of the increased recurrence rate of BPPV during the perinatal period and consider screening for underlying metabolic disturbances such as hypocalcemia or vitamin D deficiency when episodes recur [70].

The primary therapeutic challenge in managing BPPV during pregnancy lies in the logistical limitations of performing repositioning maneuvers safely and effectively, especially in the later stages of gestation [60-69]. Additionally, misdiagnosis is common as vertigo in pregnancy may be mistakenly attributed to normal physiological changes [60-69]. Further studies are needed to develop pregnancy-specific protocols and to evaluate long-term outcomes in this population [60-69].

Ménière’s disease

MD is a chronic inner ear disorder characterized by episodic vertigo, fluctuating sensorineural hearing loss, tinnitus, and aural fullness, attributed to endolymphatic hydrops - abnormal accumulation of endolymph within the membranous labyrinth [71]. Although the disease typically manifests in middle-aged adults, it can affect women of reproductive age and poses unique therapeutic challenges when present during pregnancy [72,73]. The underlying pathophysiology in pregnancy is thought to be influenced by fluid retention, increased vasopressin levels, and hormonal changes - particularly elevated estrogen and progesterone - which may alter inner ear homeostasis and exacerbate endolymphatic imbalance [72,73]. Some reports suggest that pregnancy may lead to partial symptomatic relief, possibly due to the anti-inflammatory effects of increased endogenous corticosteroids; however, other cases experience worsening of vertigo and hearing fluctuations [9].

Management of Ménière’s disease during pregnancy is complex and often requires a balance between conservative strategies and judicious pharmacologic intervention [73]. First-line management relies on dietary modification, particularly sodium restriction, aimed at reducing endolymphatic pressure [73]. Low-sodium diets (typically <1500-2000 mg/day) remain the cornerstone of preventive treatment and are considered safe during pregnancy [73]. Patients should also be advised to avoid known dietary triggers such as caffeine, alcohol, and foods rich in monosodium glutamate [73]. Ensuring adequate hydration and avoiding excessive physical stress are also general supportive measures [73].

Pharmacological options are limited due to teratogenic concerns [74]. Diuretics, such as hydrochlorothiazide or acetazolamide, are commonly used in the general population to reduce inner ear fluid volume but are generally avoided during pregnancy due to potential risks of fetal electrolyte imbalances, reduced placental perfusion, and oligohydramnios [74]. There are isolated reports of diuretic use without adverse outcomes; however, the risk-benefit profile generally does not favor their use in routine management during gestation [74].

Betahistine dihydrochloride, a histamine H1 receptor agonist and H3 receptor antagonist, is widely used in Europe as a prophylactic agent for Ménière’s disease [75]. Betahistine is classified as Category C due to the absence of controlled human studies; however, it is often continued in patients with established benefit before pregnancy under close medical supervision [75]. Its vasodilatory effect on cochlear microcirculation and modulation of vestibular nuclei makes it a rational therapeutic choice in selected cases [75].

Acute vertigo attacks are typically managed with vestibular suppressants and antiemetics. Meclizine, promethazine, and diphenhydramine may be used short-term to control severe vertiginous episodes [76]. These medications are considered relatively safe (Category B), but should be used with caution due to potential sedative effects and the possibility of masking progressive vestibular dysfunction. Importantly, these agents do not modify the course of the disease and are limited to symptomatic relief [76].

Intratympanic corticosteroid injections, such as dexamethasone, may be considered in refractory or unilateral cases [77]. This localized approach offers the theoretical advantage of reducing inflammation and immune-mediated inner ear injury with minimal systemic exposure, thereby mitigating potential risks to the fetus [77]. However, safety data specific to intratympanic therapy in pregnancy are sparse, and this option should be reserved for debilitating cases under otologic supervision [77]. Systemic steroids are generally avoided due to risks including maternal glucose intolerance, gestational hypertension, and fetal growth restriction, unless indicated [77].

To date, there is a lack of literature and insufficient scientific evidence supporting the safety or efficacy of surgical interventions such as endolymphatic sac decompression, vestibular neurectomy, or labyrinthectomy during pregnancy [77]. Similarly, intratympanic administration of gentamicin, typically reserved for refractory cases due to its vestibulotoxic properties, is contraindicated in pregnancy owing to the potential risk of fetal ototoxicity and systemic toxicity [78].

The therapeutic management of Ménière’s disease in pregnancy thus relies on conservative measures, with limited pharmacologic options applied in a highly individualized fashion [73]. Multidisciplinary care involving otolaryngologists, maternal-fetal medicine specialists, and audiologists is essential to ensure optimized maternal health while minimizing fetal exposure [73]. Future prospective studies are warranted to evaluate the safety and efficacy of current and emerging therapies for MD in pregnant patients [73].

Persistent postural-perceptual dizziness

PPPD is a chronic functional vestibular disorder characterized by persistent sensations of non-spinning dizziness, unsteadiness, or swaying that are exacerbated by upright posture, active or passive motion, and exposure to complex visual environments. PPPD is often triggered by an acute vestibular insult, a medical illness, or significant psychological stress [79]. Its pathophysiology involves maladaptive neuroplastic changes in spatial orientation networks, hypersensitivity to motion stimuli, and heightened activity in limbic and prefrontal circuits [79]. In pregnant women, hormonal fluctuations, emotional lability, fatigue, and increased physiological demands may exacerbate PPPD symptoms or unmask latent susceptibility [9]. Diagnosing PPPD in pregnancy is particularly challenging because dizziness, lightheadedness, and postural intolerance overlap with common gestational phenomena such as orthostatic hypotension, anemia, and hyperemesis gravidarum [80].

The cornerstone of PPPD treatment, particularly during pregnancy, may lie in non-pharmacological interventions, given the limited safety data on pharmacological options in this population [81,82]. VRT plays a central role and has demonstrated effectiveness in reducing disability and improving postural control [47]. VRT protocols should include habituation exercises, gaze stabilization, and dynamic balance training [81,82]. In pregnancy, these programs should be adapted to account for gestational musculoskeletal changes and patient tolerance [81,82]. Early initiation of VRT has been shown to reduce the likelihood of chronicity and dependence on pharmacologic agents [81,82].

CBT is another first-line modality, particularly effective in addressing the maladaptive thought patterns and anxiety that perpetuate PPPD symptoms [83]. CBT has shown robust efficacy in both primary anxiety and secondary dizziness-related anxiety, and its safety in pregnancy is well established [83]. Additionally, mindfulness-based stress reduction (MBSR) techniques and guided breathing exercises may improve autonomic regulation and reduce subjective symptom severity [83].

Pharmacologic interventions may be necessary in moderate to severe cases, especially when non-pharmacological approaches alone are insufficient [20]. SSRIs, particularly sertraline and escitalopram, have the most robust evidence for efficacy in PPPD and are considered relatively safe during pregnancy, especially when initiated after the first trimester [20]. Sertraline is often preferred due to its lower placental transfer and extensive use in obstetric psychiatry [20,24]. Initiation should follow a shared decision-making process with the patient, considering the risks of untreated maternal mental health conditions versus potential fetal exposure [20]. Dosage titration should be conservative, and clinical monitoring is essential [20].

Benzodiazepines, while sometimes used in non-pregnant individuals for acute anxiety or vestibular crises, are generally contraindicated during pregnancy due to risks of teratogenicity, sedation, neonatal withdrawal syndrome, and impaired fetal neurodevelopment [36]. Their use should be strictly avoided unless in emergency psychiatric contexts and under specialized care [36]. SNRIs such as venlafaxine may be considered in refractory cases but carry more complex safety profiles and should be used with caution and psychiatric supervision [84].

A major limitation in managing PPPD during pregnancy is under recognition by healthcare providers and the stigma associated with psychogenic or functional disorders [84]. Many women with PPPD report being dismissed or misdiagnosed with purely psychological causes, delaying effective intervention [85]. A multidisciplinary approach involving otoneurology, psychiatry, obstetrics, and physical therapy is crucial to ensure comprehensive care [85]. Importantly, clinicians should emphasize that PPPD is a real and treatable condition with neurobiological underpinnings, not a psychological fabrication [85].

In conclusion, the management of PPPD during pregnancy is centered on behavioral therapy and physical rehabilitation, with pharmacologic therapy reserved for persistent or functionally disabling symptoms [85]. There is an urgent need for studies evaluating treatment outcomes and safety profiles of psychotropic medications, specifically in pregnant individuals with vestibular conditions, as existing guidelines often rely on extrapolated data from the psychiatric literature.

Unilateral vestibulopathy

Unilateral vestibulopathy (UVL) is an acute peripheral vestibular syndrome that manifests as a sudden, prolonged episode of vertigo, typically lasting several hours to days, without associated hearing loss [86]. It is presumed to result from inflammation of the vestibular nerve, most likely due to reactivation of latent viral infections, especially herpes simplex virus type 1 (HSV-1) [86]. Although the incidence of UVL during pregnancy is not well characterized, physiologic and immunologic changes inherent to gestation, including immune modulation, increased cortisol levels, and altered stress responses, may predispose susceptible individuals to viral reactivation [86]. In addition, the clinical presentation may overlap with other causes of dizziness in pregnancy, including posterior circulation ischemia, vestibular migraine, and hemodynamic changes, complicating timely diagnosis [86].

The therapeutic approach to UVL in pregnancy must consider both the intensity of vertigo symptoms and the safety of pharmacologic agents [86]. In the general population, high-dose oral corticosteroids administered within 72 hours of symptom onset have demonstrated benefit in improving long-term vestibular function, presumably by reducing neural inflammation and promoting faster compensation [86,87]. However, in pregnant patients, systemic corticosteroid use is controversial [88]. Although prednisone is considered relatively safe when used short-term during the second or third trimesters, its use in the first trimester has been associated with a small increased risk of orofacial clefts in some studies [88]. Therefore, systemic steroids may be considered in pregnant women with debilitating vertigo that significantly impairs mobility, hydration, or nutrition, but only after rigorous risk-benefit assessment and preferably in consultation with obstetrics and maternal-fetal medicine [88]. If used, short courses of prednisone (1 mg/kg/day for five to seven days) followed by rapid tapering are recommended, with close monitoring of maternal glucose and blood pressure [88].

For acute symptom control, vestibular suppressants such as meclizine and promethazine may be employed judiciously [89]. These antihistamines are classified as FDA Category B and are commonly used for nausea and vertigo in pregnancy [89]. They are effective in managing acute distress during the first 24-72 hours but should be discontinued as soon as tolerable to avoid delaying central vestibular compensation [89]. Importantly, the goal in UVL management is not only symptom relief but also the promotion of neuroplastic adaptation; therefore, prolonged use of suppressants is counterproductive [89].

VRT is the mainstay of long-term recovery and is crucial in the subacute and chronic phases of UVL [39,48]. VRT protocols may include gaze stabilization exercises, balance retraining, and habituation exercises tailored to the patient’s level of physical tolerance and gestational stage [39,48]. VRT has consistently demonstrated effectiveness in reducing postural instability, enhancing functional recovery, and minimizing the risk of chronic dizziness or secondary functional disorders such as PPPD [48]. It is safe in all trimesters and should be initiated as soon as the acute vertigo subsides [48]. Starting with the vestibulo-ocular reflex (VOR) with vergence exercise can increase the gain of the VOR within one session and may create fewer symptoms as they start their exercise program [90]. Early initiation of exercise within the first two weeks results in greater VOR gain values and better functional outcomes [91]. 

Other pharmacologic agents commonly used in vestibular disorders such as benzodiazepines (e.g., diazepam, lorazepam) are not recommended during pregnancy due to well-established risks, including teratogenicity, neonatal withdrawal, and sedation [92]. Similarly, antivirals (e.g., acyclovir or valacyclovir), though used in severe HSV reactivations, have not demonstrated consistent efficacy in UVL and are generally not indicated unless there is clinical or laboratory evidence of systemic viral disease [93]. The main therapeutic challenges in managing UVL during pregnancy include diagnostic uncertainty in the context of overlapping gestational symptoms, hesitancy to use corticosteroids, and limited clinical trial data [88].

Bilateral vestibulopathy

BVP is a chronic condition characterized by bilateral impairment or loss of vestibular function, leading to symptoms such as oscillopsia, unsteadiness, particularly in darkness or on uneven ground, and postural imbalance [93]. While BVP is more commonly associated with ototoxicity (especially aminoglycosides), neurodegenerative diseases, autoimmune inner ear disorders, or idiopathic etiologies, it can also result from sequential vestibular insults such as bilateral vestibular neuritis or progressive vestibular loss in conditions like Cogan’s syndrome or bilateral Ménière’s disease [94]. Although BVP is rare in women of childbearing age, its management during pregnancy presents a unique set of clinical challenges, primarily due to safety limitations of vestibular-active medications and the potential impact of chronic instability on maternal mobility, quality of life, and fall risk [94].

There is currently no curative treatment for BVP in pregnancy [94]. Management focuses on compensatory strategies aimed at enhancing visual and somatosensory substitution, reducing fall risk, and improving functional mobility [95]. VRT is the cornerstone of treatment and has been shown to significantly improve gait stability and reduce oscillopsia in non-pregnant populations [94-96]. In pregnant patients, VRT should be adapted to their changing center of mass, ligamentous laxity, and risk of joint instability [19]. Therapy should include gaze stabilization exercises (e.g., adaptation and substitution protocols), dynamic gait training with external cueing, balance reweighting exercises, and dual-task training [94-96]. Home safety assessments and fall prevention strategies are essential, particularly in the third trimester when the risk of imbalance increases physiologically [94].

The psychosocial burden of BVP during pregnancy should not be underestimated. Persistent imbalance may limit daily activity, increase fear of falling, and contribute to emotional distress or depressive symptoms, especially in patients who are otherwise physically active or experiencing a first pregnancy [96]. Structured psychological support, including CBT or counseling, may aid in the overall adaptation process [96]. Occupational therapy can also provide tools for environmental modification and functional task adaptation during pregnancy [96].

Therapeutic and clinical challenges in the management of vestibular disorders during pregnancy

The therapeutic management of vestibular disorders during pregnancy involves a series of unique and multifaceted challenges, derived from physiological, pharmacological, diagnostic, and ethical considerations [9]. These challenges complicate not only clinical decision-making but also the development of standardized treatment protocols, given the delicate balance required between maternal well-being and fetal safety [9].

Overlapping Symptoms

One of the primary diagnostic and therapeutic challenges is the overlap between vestibular symptoms and normal physiological changes of pregnancy [9]. Nausea, orthostatic hypotension, dizziness, fatigue, and imbalance are commonly reported in healthy pregnancies, especially during the first and third trimesters [9]. These symptoms may obscure the recognition of underlying vestibular pathology, delaying diagnosis and appropriate intervention [9,47]. Conditions such as PPPD or early-stage vestibular migraine may be misattributed to gestational anxiety, hormonal fluctuations, or vasovagal instability [9,47]. 

Limited Safety Data on Pharmacological Therapies

Pharmacologic treatment of vestibular disorders in pregnancy is constrained by the lack of robust safety data for most vestibular-active medications [47]. Most medications used in vertigo management, including SSRIs, corticosteroids, antihistamines, and betahistine, are classified as FDA Category B or C, indicating limited or no controlled data in pregnant populations [10,97]. This leads to clinical uncertainty and considerable variability in prescribing practices [10,97]. Ethical barriers prevent the inclusion of pregnant individuals in randomized controlled trials, perpetuating a reliance on retrospective cohort studies, case reports, and pharmacovigilance registries, many of which are underpowered or suffer from reporting bias [97].

Contraindication or Modification of Standard Maneuvers and Procedures

Standard vestibular maneuvers and rehabilitation protocols may require pregnancy modifications [9]. For instance, canalith repositioning maneuvers such as the Epley or Semont may not be feasible in the third trimester due to physical constraints, supine hypotension syndrome, or musculoskeletal discomfort [97]. Similarly, balance training and VRT protocols must be carefully adapted to gestational biomechanics and fatigue thresholds [48,97]. This often necessitates collaboration with physical therapists experienced in both vestibular rehabilitation and pregnancy care, which may not be readily available in all clinical settings [48].

Absence of Clinical Guidelines and Standardized Protocols

There are currently no international or national clinical guidelines specifically addressing the management of vestibular disorders in pregnancy [97]. Most recommendations are extrapolated from general vertigo management guidelines or adapted from psychiatric, neurological, or obstetric sources [97].

Risks of Maternal Functional Impairment

Vestibular disorders can significantly impair mobility, balance, and daily functioning, potentially affecting prenatal care compliance, physical activity, and maternal-fetal bonding [1]. In disorders such as bilateral vestibulopathy or severe VM, the risk of falls or social withdrawal is heightened, which may increase maternal stress and reduce quality of life [1]. Despite this, vestibular dysfunction is rarely included in standard prenatal risk assessments, leaving these patients without adequate support [1].

Research Gaps and Scientific Barriers

Major knowledge gaps persist regarding the epidemiology, natural history, and long-term maternal-fetal outcomes of vestibular disorders in pregnancy [98]. The exclusion of pregnant individuals from most clinical trials limits the evidence base needed to inform safe and effective treatment strategies [98].

## Conclusions

Vestibular disorders during pregnancy require a highly individualized and trimester-sensitive therapeutic approach. The physiological changes inherent to gestation can both precipitate and exacerbate vestibular conditions, while simultaneously limiting diagnostic clarity and therapeutic options. Non-pharmacological interventions, such as vestibular rehabilitation and psychological support, remain first-line strategies throughout pregnancy, with pharmacologic agents introduced cautiously based on gestational age and symptom severity. A multidisciplinary model of care is essential to balance maternal symptom control with fetal safety. Future research must address the current evidence gaps to guide standardized, safe, and effective management protocols for this unique population.
